# Fibroblast Activation Protein Inhibitor Positron Emission Tomography Imaging in Atrial Fibrillation: Current Evidence and Future Perspectives

**DOI:** 10.31083/RCM50485

**Published:** 2026-09-10

**Authors:** Yi Xu, Huiyi Qiu, Chuxian Guo, Qianyun Wang, Yongjin Tang, Chongyi Huang, Jun Guo, Feifei Wang

**Affiliations:** ^1^School of The First Clinical Medical, University of Jinan, 510632 Guangzhou, Guangdong, China; ^2^Department of Cardiology, The First Affiliated Hospital of Jinan University, 510632 Guangzhou, Guangdong, China; ^3^Health Management Division, Shunde Hospital, Southern Medical University (The First People’s Hospital of Shunde), 528308 Foshan, Guangdong, China; ^4^Department of Nuclear Medicine, The First Affiliated Hospital of Jinan University, 510632 Guangzhou, Guangdong, China

**Keywords:** fibroblast activation protein inhibitor, positron emission tomography, arrhythmia, atrial fibrillation, myocardial fibrosis

## Abstract

Fibroblast activation protein inhibitor (FAPI) positron emission tomography/computed tomography (PET/CT) has emerged as a promising molecular imaging modality for visualizing fibroblast activation and extracellular matrix remodeling. In addition to its established role in oncology imaging, FAPI PET/CT is gaining recognition in the evaluation of cardiovascular disease. Emerging evidence suggests that this technique may provide molecular insights into atrial fibroblast activation and the development of a fibrotic substrate in atrial fibrillation. Meanwhile, related findings from other arrhythmias further support a potential link between myocardial fibroblast activation and electrophysiological remodeling. This review summarizes the current evidence on FAPI PET/CT imaging in atrial fibrillation, discusses related findings in other cardiovascular conditions, and highlights future directions for research in arrhythmia imaging.

## 1. Introduction

Cardiac arrhythmias are common clinical conditions characterized by irregular electrical activity in the heart, which are associated with significant adverse cardiovascular outcomes. Myocardial fibrosis plays a central role in their pathophysiology by disrupting conduction uniformity and promoting reentrant circuits [1]. Conventional imaging modalities, such as cardiac magnetic resonance imaging (MRI) with late gadolinium enhancement (LGE), visualize established fibrosis but cannot identify early fibroblast activation preceding irreversible structural remodeling [1,2,3].

Fibroblast activation protein (FAP) is a type II transmembrane serine protease expressed by activated fibroblasts during tissue repair and pathological fibrosis [4]. Radiolabeled FAP inhibitors (FAPI) have enabled *in vivo* visualization of fibroblast activity using positron emission tomography (PET) imaging [5]. While FAPI imaging has been extensively investigated in oncology [6], its cardiovascular applications have expanded in recent years, particularly in myocardial fibrosis regarding heart failure, myocardial infarction, and cardiomyopathies [7]. Current evidence has also suggested a potential role of FAPI PET imaging in atrial fibrillation, particularly for visualizing atrial fibroblast activation and fibrotic remodeling. This review summarizes current evidence on FAPI PET imaging in atrial fibrillation, reviews related observations from other cardiovascular conditions, and highlights future directions for applying molecular imaging to arrhythmia research.

## 2. Principles and Characteristics of FAPI PET Imaging Techniques

The fundamental principle of FAPI PET imaging is the use of radiolabeled FAPI molecular probes to target activated fibroblasts, which play a critical role in cardiovascular diseases, particularly in fibrotic processes [8]. As an efficient molecular tracer, FAPI binds to fibroblast activation protein expressed on the surface of fibroblasts and enables visualization by PET scanning [9]. Radiopharmaceuticals targeting FAP include several tracers, such as FAPI-04, FAPI-46, and ^18^F-labeled FAPI derivatives. Different tracers may vary in radionuclide half-life, production feasibility, biodistribution, uptake time, blood-pool activity, clearance, and tissue retention, all of which are relevant to cardiac imaging. ^68^Ga-labeled tracers have been widely used in early clinical studies, but their relatively short physical half-life may limit centralized production and long-distance distribution [5]. In contrast, ^18^F-labeled tracers offer practical advantages for wider clinical distribution because of the longer half-life of ^18^F. However, cardiac-specific performance remains tracer- and disease-dependent, and further validation is needed before any tracer can be considered optimal for cardiac or arrhythmia imaging.

FAPI PET imaging has shown considerable potential in cardiovascular disease by enabling early and noninvasive assessment of myocardial fibrosis and tissue remodeling [10,11,12]. Through specific targeting of activated fibroblasts, FAPI tracers may visualize fibrotic activity before irreversible structural changes become evident on conventional imaging methods, offering insight into disease progression and potential treatment decision-making [12]. Compared with conventional imaging modalities such as MRI or computed tomography (CT), FAPI PET may provide valuable complementary molecular information for evaluating fibrotic activity in conditions including atrial fibrillation, atherosclerosis, and coronary artery disease [13,14].

## 3. Applications of FAPI PET Imaging in Arrhythmia

Myocardial fibroblast activation has been implicated in the development of arrhythmogenic fibrosis, particularly in atrial fibrillation (AF) and ventricular tachycardia [15]. Activated fibroblasts and myofibroblasts contribute to extracellular matrix deposition, conduction heterogeneity, and reentry substrate formation [16]. Imaging fibroblast activation with FAPI PET imaging thus allows noninvasive detection of active fibrogenesis that precedes irreversible scar formation, providing mechanistic insight into arrhythmia substrates. The standard workflow for ^18^F-FAPI PET/CT imaging is shown in Fig. 1.

**Fig. 1.**
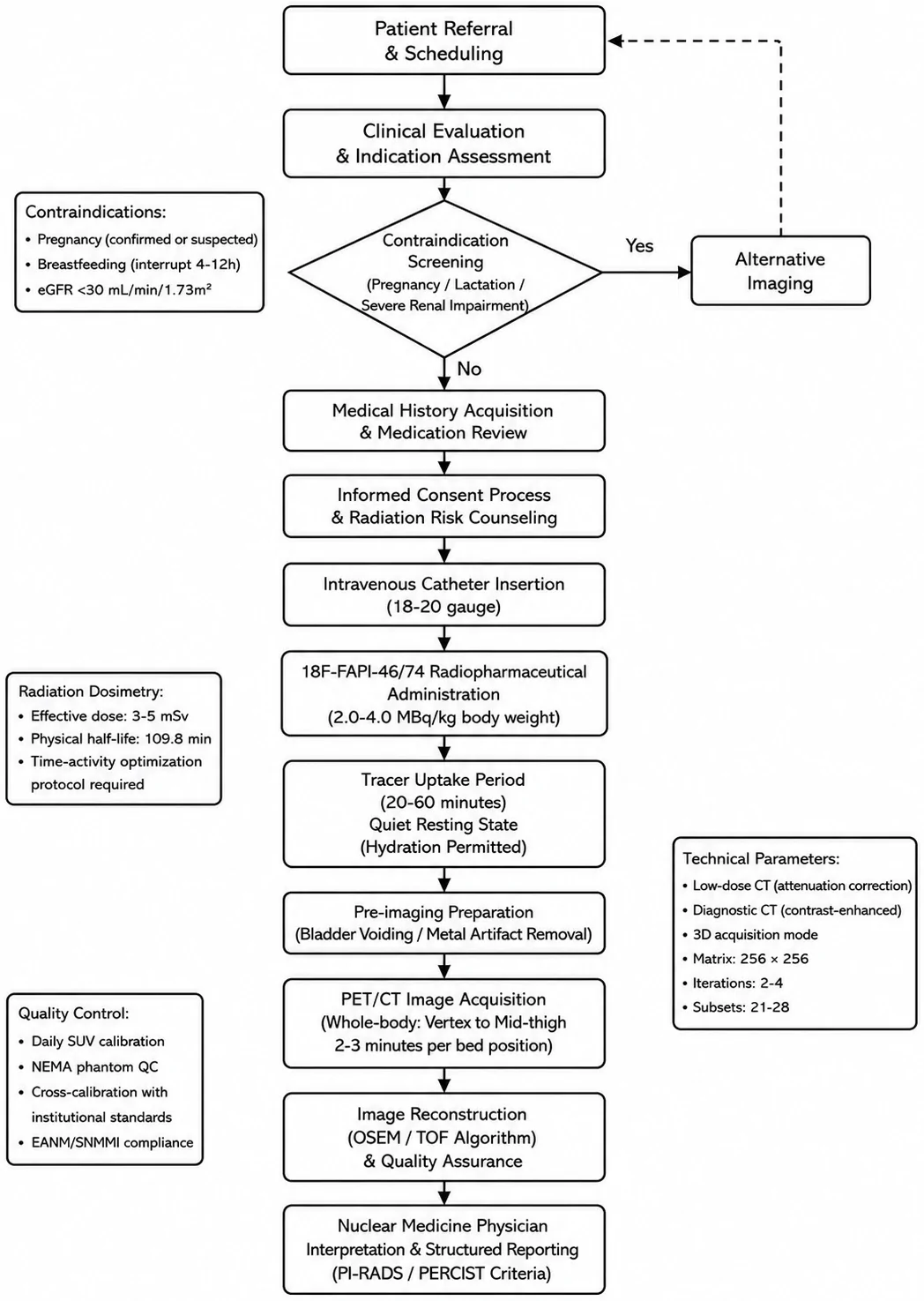
**Standardized workflow for ^18^F-FAPI PET/CT imaging**. FAPI, fibroblast activation protein inhibitor; PET/CT, positron emission tomography/computed tomography; ^18^F, fluorine-18; eGFR, estimated glomerular filtration rate; CT, computed tomography; OSEM, ordered-subset expectation maximization; TOF, time-of-flight; PI-RADS, Prostate Imaging Reporting and Data System; PERCIST, PET Response Criteria in Solid Tumors; SUV, standardized uptake value; NEMA, National Electrical Manufacturers Association; QC, quality control; EANM, European Association of Nuclear Medicine; SNMMI, Society of Nuclear Medicine and Molecular Imaging; 3D, three-dimensional.

Delayed late gadolinium enhancement cardiac magnetic resonance (LGE-CMR) is regarded as the “gold standard” for non-invasive evaluation of myocardial fibrosis. However, it requires gadolinium contrast agent administration, imposes limitations on patients with renal insufficiency, exhibits low sensitivity for diffuse fibrosis, involves high costs, and requires prolonged scanning time. Recent studies [17,18,19] have explored FAPI PET as a method to characterize fibrosis-related mechanisms in arrhythmia, with growing clinical and preclinical evidence supporting its application (Fig. 2).

**Fig. 2.**
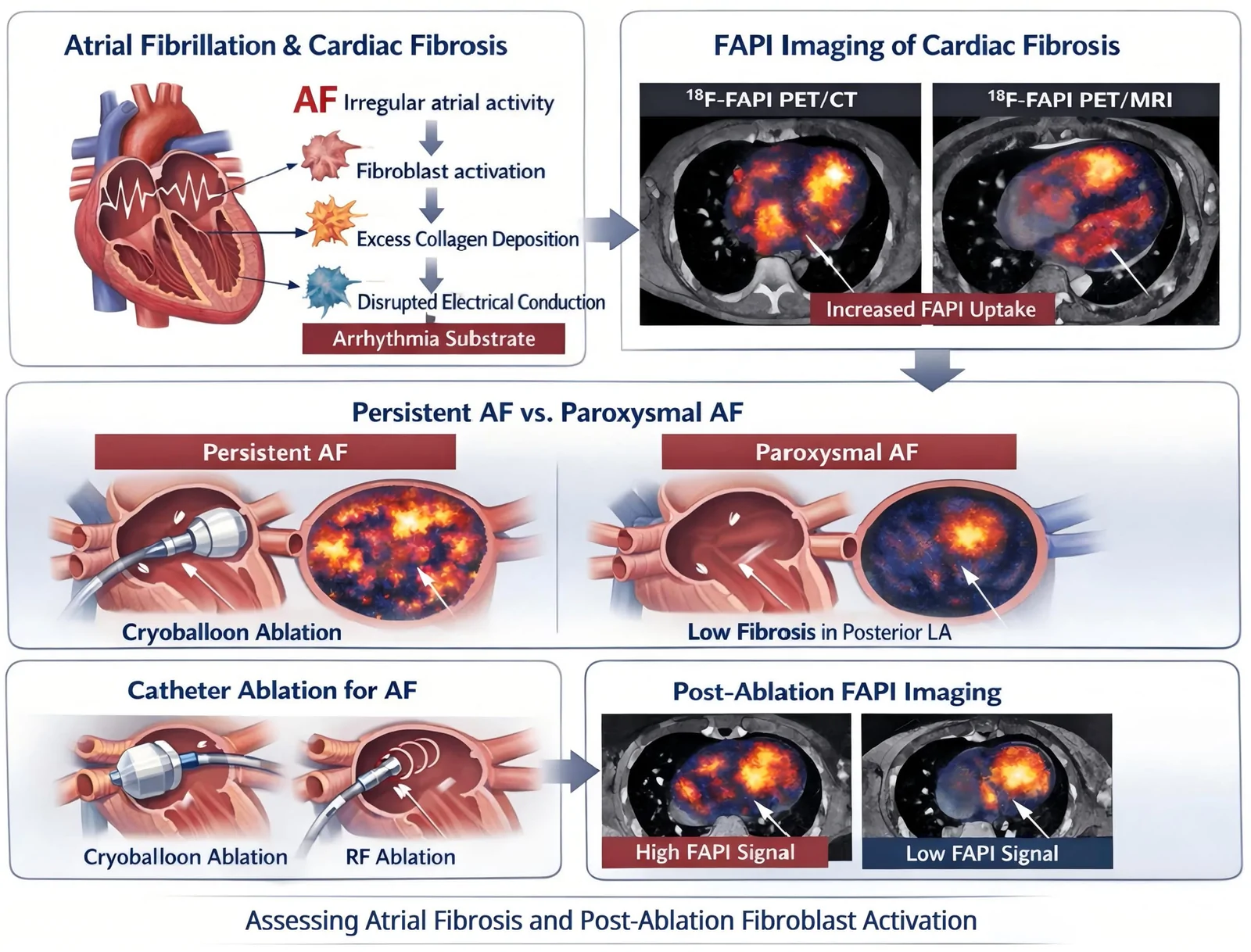
**Assessing atrial fibrosis & predicting AF recurrence**. This schematic illustrates the relationship between AF, fibroblast activation, extracellular matrix remodeling, and atrial fibrotic substrate formation. Increased FAPI uptake may reflect active fibroblast activation and can be visualized by PET/CT, providing a molecular imaging perspective on atrial remodeling in AF. These images were provided and edited by the First Affiliated Hospital of Jinan University. AF, atrial fibrillation.

### 3.1 Atrial Fibrillation

AF represents the most extensively studied type of arrhythmia using FAPI PET imaging. AF is closely linked to atrial fibrosis, which contributes to electrical and structural remodeling, promoting reentry circuits and conduction heterogeneity [20,21]. Late gadolinium enhancement (LGE) cardiovascular magnetic resonance (CMR) imaging has been widely studied as a noninvasive method for evaluating left atrial structural remodeling in AF. However, its use for routine clinical delineation of atrial fibrosis remains limited. Reliable quantification of left atrial fibrosis by CMR is technically challenging because of the thin atrial wall, partial-volume effects, motion and rhythm-related artifacts, variable image quality, and the lack of universally standardized segmentation and thresholding methods. These limitations have restricted external validation and reproducibility across centers and have contributed to ongoing skepticism regarding the accuracy of CMR-based atrial fibrosis assessment [22,23]. Traditional MRI techniques mainly visualize established extracellular matrix expansion or scar and cannot directly identify early fibroblast activation preceding irreversible structural remodeling. In this context, recent advances in FAPI PET imaging have made it possible to visualize activated fibroblasts *in vivo*, thereby providing a potentially complementary molecular imaging tool for assessing active atrial fibrotic remodeling.

In a proof-of-concept study, Li et al. [17] found significantly higher ^18^F-FAPI uptake in the atria of patients with persistent AF as compared to controls. A total of 64.3% of AF patients demonstrated increased atrial FAPI uptake, which correlated with collagen deposition and FAP expression confirmed by immunohistochemistry. This finding supports the potential of FAPI as a biomarker for atrial fibrotic burden.

Furthermore, a study with FAPI PET-MRI showed that the degree of fibrosis of the left atrial posterior wall in persistent AF patients was significantly higher than in paroxysmal AF patients [18]. The positive ratio in this region exhibited high diagnostic performance for distinguishing AF subtypes (area under the curve, AUC = 0.991). Although this result was derived from a relatively small single-study cohort and may be vulnerable to overfitting or optimism bias without independent external validation, it suggests that left atrial posterior wall fibrosis may represent a key pathological basis and histological marker of AF progression.

Representative FAPI PET images from our center are shown in Fig. 3 to illustrate the heterogeneity of atrial fibroblast activation in patients with AF. These images demonstrate visually recognizable uptake patterns, including focal uptake, diffuse patchy uptake, and more extensive patchy uptake, which are presented in relation to three major clinical AF phenotypes: paroxysmal, persistent, and long-standing persistent AF. Overall, these are consistent with previously reported observations and suggest that FAPI PET imaging may provide additional descriptive information on atrial fibroblast activity across different AF phenotypes. However, these cases are provided for illustrative purposes only and do not represent a formal quantitative cohort analysis. Therefore, further studies are still needed to draw any definitive conclusions regarding the relationship between specific atrial FAPI uptake patterns and AF phenotypes.

**Fig. 3.**
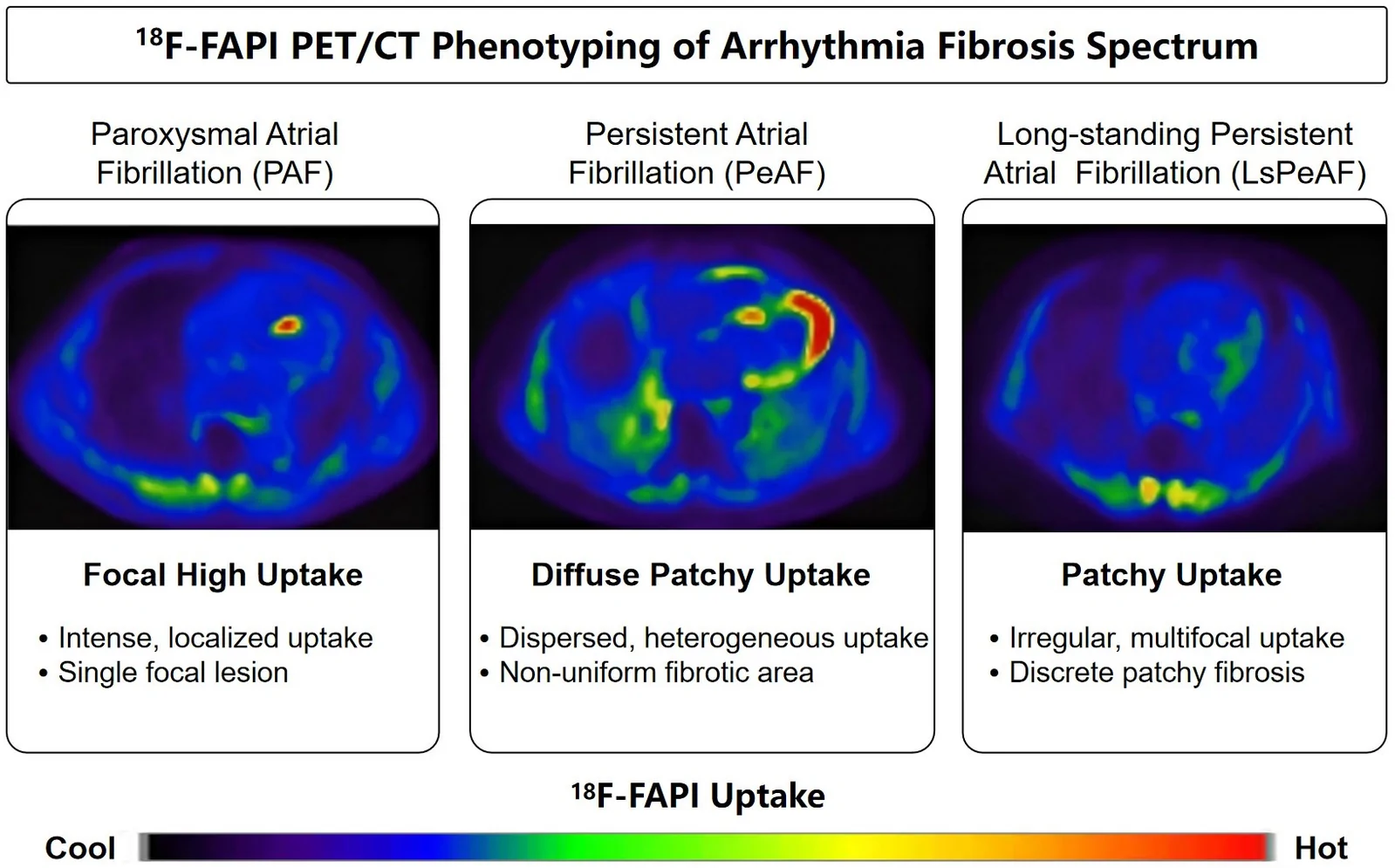
**Representative ^18^F-FAPI PET/CT uptake patterns in atrial fibrillation**. Representative images illustrate visually recognizable atrial FAPI uptake patterns across three major clinical AF phenotypes: paroxysmal, persistent, and long-standing persistent AF. These examples highlight the heterogeneity of atrial fibroblast activation and provide visual context for the potential use of FAPI PET/CT in AF-related remodeling. Further quantitative validation is warranted. Images were provided and edited by the First Affiliated Hospital of Jinan University. The use of patient images was approved by the institutional ethics committee, and all images were de-identified.

Catheter ablation is a minimally invasive procedure that treats AF by eliminating abnormal intracardiac electrical signal. Pulmonary vein isolation (PVI) is the foundation of all ablation procedures [24]. However, the procedure itself may cause injury, which could affect AF recurrence. Kupusovic et al. [19] have demonstrated that in 12 patients after pulmonary vein isolation that ^68^Ga-FAPI PET imaging detected focal uptake around the pulmonary veins in 83.3% of patients. Uptake intensity and extent were greater after cryo-ablation than radiofrequency ablation [19], suggesting that FAPI PET may visualize procedure-related fibroblast activation after ablation. However, this comparison was based on a small, non-randomized cohort. Thus the clinical significance of post-ablation FAPI uptake remains uncertain. In particular, no available data have demonstrated that FAPI PET can predict AF recurrence after ablation.

Collectively, these findings suggest that FAPI PET imaging may provide complementary molecular information on atrial fibroblast activation in AF and post-ablation tissue remodeling. At present, however, its role in ablation planning, follow-up imaging, or recurrence prediction remains investigational. Future studies with standardized imaging protocols, quantitative PET parameters, electroanatomic mapping correlation, and long-term follow-up are needed to validate the prognostic value of FAPI PET imaging.

### 3.2 Other Arrhythmias: Indirect Evidence and Future Direction

Compared with atrial fibrillation, evidence linking FAPI PET imaging to other non-AF arrhythmias remains indirect. To date, no published studies have yet evaluated FAPI PET specifically for ventricular arrhythmia. However, it has demonstrated myocardial uptake patterns consistent with fibroblast activation and interstitial remodeling in several other cardiovascular diseases. These processes are relevant to adverse structural remodeling and may contribute to abnormal electrical substrate [25], but their direct relationship with ventricular arrhythmia endpoints has not been established.

In patients with coronary artery disease and cardiomyopathy, FAPI PET highlights regions of fibroblast activation and interstitial remodeling, both of which have been considered possible triggers for ventricular tachyarrhythmias. For instance, Luo et al. [26] study with ^68^Ga-FAPI PET imaging in patients with coronary artery disease revealed localized myocardial uptake that correlated with left ventricular dysfunction and abnormal wall motion, indicating fibroblast activation beyond scarred areas. Moreover, Li et al. [27] observed elevated FAPI uptake in a series of patients with hypertrophic obstructive cardiomyopathy, which was found to correspond with fibroblast infiltration in septal myocardium histopathologically. A study in dilated cardiomyopathy patients using ^18^F-FAPI-42 PET also revealed diffuse myocardial fibroblast activation correlating with histopathological collagen deposition and function [28]. These findings suggest that FAPI PET imaging may hold potential value for future application in the assessment and risk stratification of ventricular arrhythmias.

To our knowledge, no study has specifically evaluated the role of FAPI PET in other types of arrhythmias. However, studies in chronic disease populations have suggested that abnormal myocardial FAPI uptake may correlate with electrophysiological alterations, further leading to arrhythmias. Wang et al. [29] conducted a prospective imaging study of patients with chronic liver disease and found abnormal myocardial ^18^F-FAPI PET uptake was associated with QRS voltage and duration on electrocardiography, suggesting a possible association between myocardial fibroblast activity and conduction abnormalities. In patients with systemic sclerosis-related myocardial fibrosis, increased ^68^Ga-FAPI-04 uptake was observed in conjunction with electrocardiography (ECG) abnormalities and documented arrhythmias, indicating that elevated fibroblast activity might correspond with clinically relevant electrical remodeling [30]. However, these studies did not specifically analyze arrhythmia endpoints, nor did they establish whether FAPI uptake predicts clinically relevant rhythm outcomes.

Taken together, available non-AF evidence should be viewed as supportive of future research rather than as direct clinical evidence. These observations suggest that FAPI PET may help explore the relationship between myocardial fibroblast activation, structural remodeling, and electrical abnormalities, but dedicated prospective studies are required before any role in ventricular or other arrhythmia evaluation can be defined. The key studies discussed above are summarized in Table 1 (Ref. [8,17,18,19,26,27,28,29,30]) to highlight the current scope of evidence and the remaining gaps in clinical validation.

**Table 1.** **Summary of key clinical and preclinical FAPI PET studies cited in this review**.

Study	Design	Sample size	Tracer/modality	Endpoints	Main findings
Kosmala et al. [8], 2023	Retrospective clinical imaging study	69 patients	^68^Ga-FAPI PET/CT	Arterial FAPI uptake; plaque burden; cardiovascular risk factors	Arterial FAPI uptake was associated with calcified plaque burden but showed no consistent association with conventional cardiovascular risk factors.
Li et al. [17], 2023	Proof-of-concept animal and clinical study	5 AF beagle models; 28 AF patients; 10 healthy controls	^18^F-FAPI PET/CT	Atrial FAPI uptake; FAP expression; collagen markers	Atrial FAPI uptake was increased in AF and correlated with FAP/collagen markers.
Zhang et al. [18], 2025	Prospective observational PET-MRI study	78 AF patients; 49 healthy volunteers; 36 surgical patients with 124 tissue samples	^18^F-AlF-FAPI PET-MRI	Regional atrial fibrosis; AF stage; histology	Atrial FAPI uptake increased with AF progression; LAPW showed prominent fibrosis.
Kupusovic et al. [19], 2022	Proof-of-concept post-ablation study	12 PVI patients; 5 controls	^68^Ga-FAPI PET/CT	PV-region uptake after ablation	Post-ablation PV-region uptake was detected, more pronounced after cryoablation.
Luo et al. [26], 2025	Prospective PET-MRI study	42 CHD patients after PCI	^68^Ga-FAPI PET-MRI	Myocardial uptake; LGE; wall motion	FAPI uptake extended beyond LGE and was associated with wall motion abnormality.
Li et al. [27], 2024	Case series with histology	2 HOCM patients	^18^F-FAPI PET/CT	Septal uptake; surgical histology	Septal uptake corresponded to activated fibroblasts on tissue analysis.
Liang et al. [28], 2025	Clinical PET/CT study with CMR and histology	19 DCM patients; 14 with CMR; 4 with transplant pathology	^18^F-FAPI-42 PET/CT	Fibroblast activation; CMR; collagen deposition	FAPI uptake reflected myocardial fibroblast activation and correlated with pathology.
Wang et al. [29], 2025	Prospective clinical imaging study	46 chronic liver disease patients	^18^F-FAPI PET/CT	Myocardial uptake; ECG; liver fibrosis markers	Myocardial uptake was associated with ECG parameters and liver fibrosis markers.
Treutlein et al. [30], 2023	Proof-of-concept clinical study	6 SSc-MF patients; 8 SSc without MF; 5 controls	^68^Ga-FAPI-04 PET/CT	Myocardial uptake; CMR; biopsy; ECG findings	Uptake was increased in SSc-MF and higher with arrhythmias, NT-proBNP, and LGE.

AF, atrial fibrillation; FAP, fibroblast activation protein; LAPW, left atrial posterior wall; PVI, pulmonary vein isolation; PV, pulmonary vein; CHD, coronary heart disease; PCI, percutaneous coronary intervention; LGE, late gadolinium enhancement; HOCM, hypertrophic obstructive cardiomyopathy; DCM, dilated cardiomyopathy; CMR, cardiac magnetic resonance; ECG, electrocardiogram; SSc-MF, systemic sclerosis-related myocardial fibrosis; NT-proBNP, N-terminal pro-B-type natriuretic peptide.

### 3.3 Technical Limitations of Cardiac FAPI PET

Despite its potential use, several limitations should be considered when applying FAPI PET to cardiac imaging. First, FAPI uptake is not entirely specific for mature fibrosis. Increased uptake may occur in inflammatory, reparative, degenerative, or wound-healing conditions, and therefore cardiac uptake should be interpreted together with clinical context and anatomic imaging [31,32]. Second, FAPI PET quantification may vary in different patients according to tracer type, injected activity, uptake time, scanner system, reconstruction method, blood-pool activity, and renal clearance. At present, no standardized uptake value (SUV) threshold has been validated for defining abnormal atrial FAPI uptake or distinguishing active fibroblast activation from inflammatory or reparative responses [33].

Moreover, because PET has lower spatial resolution than CT or MRI, partial-volume effects may impair the detection and quantification of tracer uptake in small or moving structures such as the atrial wall. This may lead to underestimation, blurring, or contamination from adjacent blood-pool or myocardial signal [34]. Motion correction, ECG or respiratory gating, careful co-registration with CT or MRI, and standardized segmentation strategies are therefore particularly important for atrial applications. Finally, direct head-to-head studies comparing FAPI PET with LGE-MRI, electroanatomic mapping, histology, and clinical outcomes in the same arrhythmia populations are still needed. Therefore, FAPI PET should currently still be regarded as a complementary research tool rather than a stand-alone method for clinical decision-making in arrhythmia management.

## 4. Conclusion

FAPI PET/CT imaging represents a novel molecular approach for the noninvasive assessment of myocardial fibroblast activation in arrhythmia-related remodeling. By directly visualizing fibroblast activity rather than established fibrosis, it provides complementary insights beyond conventional imaging modalities. In atrial fibrillation, current evidence suggests its potential value in detecting atrial fibroblast activation and characterizing fibrotic substrate formation. Related findings from other cardiovascular conditions remain early and hypothesis-generating, particularly outside the atrial fibrillation setting.

Future studies should establish standardized imaging and quantitative protocols, integrate FAPI PET with CMR and electroanatomic mapping, and prospectively evaluate its relationship with clinical outcomes. At present, FAPI PET should be regarded as a promising tool for studying atrial fibrillation and related cardiovascular remodeling rather than a validated clinical method for arrhythmia management.
